# Supersaturated oxygen therapy using radial artery access to prevent left ventricular remodeling after anterior ST-segment elevation myocardial infarction: a randomized, controlled trial

**DOI:** 10.1016/j.ahjo.2025.100556

**Published:** 2025-05-28

**Authors:** Francis R. Joshi, Maria Petty, Yen Wing Ng, Hamish Elliott, Ross Anderson, Douglas Gordon, Rebecca Hanna, Robert Sykes, Shaun Leonard, Andrew Morrow, Dylan Tan, Anna Kamdar, Ramu Perumal, Jeffrey L. Creech, Peter Kellman, Paul Welsh, Alex McConnachie, Colin Berry

**Affiliations:** a NHS Golden Jubilee National Hospital, Clydebank, United Kingdom; b ZOLL Medical Corporation, Chelmsford, MA, United States; c National Heart Lung and Blood Institute, Bethesda, MD, United States; d British Heart Foundation Glasgow Cardiovascular Research Centre, University of Glasgow, Glasgow, United Kingdom; e Robertson Centre for Biostatistics, University of Glasgow, Glasgow, United Kingdom

**Keywords:** Supersaturated oxygen, STEMI, Transradial, Heart failure

## Abstract

**Background:**

Novel strategies to limit the size of infarction and prevent adverse remodeling and heart failure in patients following acute ST-segment elevation myocardial infarction (STEMI) are lacking. Supersaturated oxygen (SSO_2_) therapy is approved for patients presenting within 6 h of onset of anterior STEMI using femoral artery access. The feasibility of SSO_2_ therapy via radial access is unknown. A more detailed understanding of the effect of therapy is needed.

**Objectives:**

To assess the primary outcome, defined as the within-participant change in the plasma concentration of NT-proBNP measured at baseline, 24 h, 2–5 days and 3-months post-MI.

**Design:**

Prospective, randomized, controlled, blinded, endpoint (mechanistic, PROBE) clinical trial.

**Randomized, controlled trial:**

After primary PCI, eligible participants will be blinded and randomized 2:1 to either 1 h of SSO_2_ therapy using radial artery access and intravenous glycoprotein IIbIIIa inhibitor therapy or a control (sham) procedure involving wrist manipulation in addition to standard care. The primary outcome is the within-participant change in the plasma concentration of NT-proBNP as detailed above. Secondary outcome assessments include coronary microcirculatory function, infarct size, microvascular obstruction, myocardial hemorrhage, left ventricular remodeling, myocardial blood flow, quality of life (EQ-5D-5L), Kansas City Cardiomyopathy Questionnaire (KCCQ) and the Duke Activity Status Index. Patient reported experience measures (PREMS) are an exploratory outcome. Health and economic outcomes will be assessed using electronic healthcare records.

**Value:**

The study will test the feasibility of radial artery access, provide mechanistic data and inform a larger multicenter trial powered to detect treatment effects on clinical endpoints.

Clinicaltrials.gov: NCT06662890

## 1 Background

Acute ST-segment elevation myocardial infarction (STEMI) leads to transmural myocardial infarction, left ventricular systolic dysfunction and adverse left ventricular remodeling. The duration of myocardial ischemia, quantified as being the time from symptom onset to reperfusion, correlates with the size of infarction and the potential for myocardial salvage [1,2]. When the ischemic time extends beyond 6 h, the percentage of salvaged myocardium reduces such that the size of infarction may approximate the volume of jeopardized myocardium (area-at-risk) [1].

Supersaturated oxygen (SSO_2_) is a novel therapy to enhance myocardial salvage and limit infarct size [3]. SSO_2_ involves an extracorporeal circuit including an oxygen chamber that increases the achieved partial pressure of oxygen (PO_2;_ normal 75–100 mmHg) to between 760 and 1000 mmHg. Hyperoxygenated blood is returned into the left main coronary artery and hence the ischemic myocardium supplied by the infarct-related artery using a standard coronary catheter. Prior studies have involved femoral artery access. Theoretically, hyperoxygenated blood should be provided immediately to enhance salvage of ischemic myocardium [4]. The duration of effect on myocardial metabolism and translation to recovery of contractile function are less well established.

In the Acute Myocardial Infarction With Hypoxemic Therapy (AMIHOT)-1 (*n* = 269 including 105 participants with anterior STEMI, symptoms ≤6 h duration) and AMIHOT-2 (*n* = 301 participants) randomized, controlled clinical trials, SSO_2_ therapy via femoral artery access for 1 h after primary percutaneous coronary intervention (PCI) for anterior STEMI was shown to reduce the size of infarction as assessed using Tc-99 m-sestamibi single-photon emission computed tomography (SPECT) imaging [4]. In AMIHOT-2, among 281 randomized patients with cardiac SPECT imaging at 14-days post-MI, the median (interquartile range) infarct size was 26.5 % (8.5 %, 44 %) in the control group compared with 20 % (6 %, 37 %) after SSO_2_ therapy. The pooled adjusted infarct size was 25 % (7 %, 42 %) in controls compared with 18.5 % (3.5 %, 34.5 %) after SSO_2_ therapy (*p* = 0.02; Bayesian posterior probability of superiority, 96.9 %). Thirty-day rates of major adverse cardiovascular events (MACE) in the SSO_2_ group were noninferior to the control group [5].

Observational studies have added clinical evidence for the safety of the therapy using femoral artery access [6,7]. ZOLL Circulation Inc. have received FDA and CE Mark approval for SSO_2_ therapy in patients presenting with anterior STEMI within 6 h after symptom onset. However, adoption of this therapy is limited by the design for use of femoral artery access. Furthermore, there are no data on the effects of SSO_2_ therapy on coronary microvascular function, microvascular obstruction, intramyocardial hemorrhage or left ventricular remodeling, or in patients treated with a contemporary P2Y12 inhibitor, such as prasugrel. There are no data for procedures using radial artery access, as is recommended in guidelines for primary PCI, for the extracorporeal circuit, and no data from randomized, controlled trials involving this approach, and despite the results of the AMIHOT-2 trial, SSO2 therapy is not mentioned in contemporary guidelines.

### 1.1 Rationale

Anterior STEMI is a life-threatening emergency associated with large infarction and a consequent risk of heart failure. Timely reperfusion with primary PCI is the standard of care for STEMI [8] but development of new therapies to limit the size of infarction, reduce the risk of adverse remodeling and prevent heart failure has proved challenging. No new effective therapy has been licensed for use after myocardial infarction since eplerenone twenty years ago [9]. Recent clinical trials of adjunctive reperfusion therapies (including a trial of intracoronary alteplase) have proved ineffective [[10], [11], [12]]. In response, novel approaches are being investigated, including device-based therapy [13] and intra-coronary cooling [14].

SSO_2_ therapy has shown promise in the AMIHOT trials [4,5] and the therapy is approved for use in patients with anterior STEMI within 6 h of onset of symptoms. Currently, however, the extracorporeal circuit for SSO_2_ therapy is made using femoral arterial access. However, radial artery access is recommended in clinical guidelines [8], with evidence for both reduced access site bleeding and mortality [15,16].

The study has two main objectives. The first objective is to provide preliminary evidence of efficacy for radial-based SSO₂ therapy post-MI and the second objective is to assess feasibility. Specifically, our primary clinical/physiological outcome measure is NT-proBNP, while the primary feasibility objective is to determine whether radial-based SSO₂ is achievable and safe without major bleeding or vascular complications. Secondary aims include, first, to undertake a detailed mechanistic evaluation of the effects of SSO_2_ therapy, using a combination of validated surrogate biomarkers, intracoronary physiology, and cardiovascular magnetic resonance imaging and, finally, to assess patient reported outcome measures (PROMS) and patient reported experiences measures (PREMS).

## 2 Methods

### 2.1 Study design

This is a prospective, randomized, controlled, blinded, endpoint (mechanistic, PROBE) clinical trial. The study design was informed by patient and public involvement at the NHS Golden Jubilee hospital and the research ethics service.

The rationale is to generate novel clinical data for supersaturated O_2_ therapy using exclusively radial artery access including feasibility (Table 1) and the effects on cardiac biomarkers using a randomized, controlled trial with measures to mitigate bias, including blinding with a sham experience, and blinded outcome assessments (Table 1). Therefore, the aims are for study participants, attending clinicians and outcome assessors to be blind to treatment group assignment. The effectiveness of blinding will be prospectively assessed.

**Table 1:** Secondary study objectives.

Objective	Methodology
Feasibility defined as a completed SSO2 therapy	Study design
Coronary angiogram at the end of supersaturated O2 therapy	Assessment of TIMI Coronary Flow Grade, angiographic Blush Grade, Frame Count and Thrombus Grade
ECG	Assess % ST-segment resolution on the 12- lead ECG (pre- vs. end of supersaturated O2 therapy 60 min post-reperfusion with primary PCI)
Coronary microvascular function	Assess measures of culprit artery microvascular function including the index of microvascular resistance (IMR), vasodilator capacity reflected by coronary flow reserve (CFR), resistance reserve ratio (RRR) and microvascular resistance reserve (MRR) before and after supersaturated O2 therapy, or sham control to permit between group comparisons.
Infarct characteristics	Assess myocardial infarct characteristics including infarct size, microvascular obstruction, myocardial hemorrhage, and myocardial salvage using CMR imaging 2–5 days and 3 months post-MI.
Left ventricular remodeling	Assess LV ejection fraction and LV remodeling, including LV end-systolic volume index and end-diastolic volume index, over 3 months using CMR imaging. Assess remote zone characteristics including native T1 (ms) and extracellular volume
Myocardial blood flow	Assess blood flow within myocardial segments subtended by the infarct-related artery at 3 months using stress CMR imaging.
Biomarkers	Assessment of circulating biomarkers over time (baseline (before coronary angiogram, pre-PCI, pre-randomization, pre-SSO_2_), 75 min (post-SSO_2_), 24 h ± 4 h, 2–5 days and at 3 months).
Health status	Assess health-related quality of life using patient reported outcome measures during follow-up, including the EQ-5D-5L, Duke Activity Status Index and Kansas City Cardiomyopathy questionnaires
Feasibility	Feasibility will be reflected by enrolment rates, procedure completion, adverse events, and patient reported experiences measures (PREMS) using a customized questionnaire.
Safety	Assessment of peri-procedural and post-procedural events as well as those during follow-up
Clinical outcomes	Assessment of clinical outcomes over 12 months and longer-term follow-up including data linkage

### 2.2 Intervention

Supersaturated O_2_ (SSO_2_) therapy immediately following standard care primary PCI using exclusively radial artery access.

### 2.3 Control

Control (sham) procedure in addition to standard care primary PCI.

### 2.4 Primary objective

To assess within-participant changes in plasma concentration of N-terminal pro-B-type natriuretic peptide (NT-proBNP) measured at baseline (prior to primary PCI for acute anterior STEMI), and at 24 h, 2–5 days, and 3-months after primary PCI.

### 2.5 Primary outcome

The plasma concentration of NT-proBNP at baseline (prior to primary PCI), and at 24 h, 2–5 days, and 3 months after primary PCI.

### 2.6 Secondary objectives

The prespecified secondary objectives are listed in Table 1.

### 2.7 Implementation

The study design involves prospective enrolment of patients with a diagnosis of acute anterior STEMI presenting within 6 h of symptom undergoing primary PCI. The protocol begins after primary PCI; the timepoint for randomization (time zero) is after primary PCI. The consent process will involve an initial witnessed verbal informed consent in the catheter laboratory during and/or at end of the primary PCI as felt to be appropriate by the attending clinician and before any change in the clinical workflow. Only after informed consent is given would any study related activities (including randomization) be undertaken.

Patients who fulfil the eligibility criteria and who have given witnessed informed verbal consent will be randomly assigned (2:1) to the intervention group or the control group.

The Patient Information Sheet and Informed Consent form for this study will be provided to patients and carers on the ward. If the patient is still happy to participate then written informed consent will be obtained. Study assessments will involve blood samples, cardiovascular magnetic resonance scans, health status questionnaires, and electronic case record linkage. If the patient is eligible to participate and agrees to have electronic health record linkage follow-up but no other assessments or procedures, then consent will be invited for electronic health record follow-up only (no visits).

### 2.8 Setting

A tertiary care cardiothoracic center in the West of Scotland, United Kingdom. The NHS Golden Jubilee hospital provides primary PCI for approximately 650 patients per annum.

### 2.9 Population

All-comers referred for primary PCI for acute anterior STEMI.

### 2.10 Eligibility

The inclusion criteria are: age ≥18 years, ischemic time ≤ 6 h from symptom onset, acute anterior STEMI with infarct-related left anterior descending coronary artery TIMI flow grade 0–2 at initial angiography, infarct-related left anterior descending coronary artery TIMI flow grade 2–3 at the end of primary PCI, radial artery access and partial pressure of oxygen (PaO_2_) >80 mmHg (10.7 kPa).

The exclusion criteria are: proximal coronary artery stenosis that restricts blood flow with the SSO_2_ catheter in place, coronary dissection or perforation not treated with a stent at the completion of primary PCI, moderate or severe heart valve stenosis or insufficiency, pericardial disease, non-ischemic cardiomyopathy, known pregnancy, cardiogenic shock, contra-indication to systemic anticoagulation, acute mechanical complication (ventricular septal rupture, pseudoaneurysm, mitral regurgitation), hemoglobin <10 g/dL, major bleeding or major surgery within the past two months, contra-indication to CMR (severe claustrophobia, metallic foreign body) and/or a lack of witnessed verbal consent.

### 2.11 Enrolment, consent and randomization

Consecutive STEMI patients will be screened following referral to the CCU and on arrival at the hospital. Patients will be considered eligible according to the presence of inclusion criteria and absence of exclusion criteria. Screening will be performed by the attending cardiologist and/or research nurse; the final decision as to whether a patient is eligible to be included in the study will be made and documented by the treating cardiologist. Anterior STEMI patients will be assigned a screening log number. Baseline characteristics and the reason(s) for not being included will be recorded in a screening log database; no follow-up information will be recorded. Patients who are screened and who give informed consent will be recorded in an electronic case report form (eCRF). Patients who are initially eligible but who subsequently become ineligible (such as on angiographic grounds) will not proceed in the study and will become screen failures. Rarely, patients may be treatment allocation failures (such as for logistical reasons); their data will still be recorded in the eCRF.

Because of the need for emergency treatment, delays to obtain full written informed consent in the standard way is not appropriate. Based on experience in prior studies, initial verbal consent is proposed. This approach is in line with contemporary evidence [17], feedback from experts with lived experience and international practice guidelines [8,17]. Patients both fulfilling all inclusion criteria and no exclusion criteria and sufficiently able to understand information about the study (including the potential for benefit and known risks) would be eligible to participate and invited to give verbal informed consent by the attending cardiologist performing primary PCI.

Discussion of the study will take place in the presence of catheter laboratory staff and will cover all points listed in an approved Short Patient Information Sheet. If the patient agrees to participate, then a form to this effect will be signed by the attending interventionalist. Eligibility will be reviewed during primary PCI, reaffirmed at the end of the primary PCI and documented by a study clinician in the medical notes and/or eCRF.

On returning to the ward after completion of primary PCI and study assessments in the catheter laboratory, the patient is to be provided with a full Patient Information Sheet and invited to provide written consent. This must be obtained within 24 h of admission or prior to transfer to base hospital if planned. When this is not possible (perhaps because of clinical deterioration), consent may be delayed and obtained when this becomes feasible. No further study assessments can be performed until written informed consent is obtained.

### 2.12 Primary PCI

This is to be performed with radial arterial access. Antiplatelet therapy and systemic anticoagulation with unfractionated heparin as recommended by clinical guidelines [8]. The target activated clotting time (ACT) during primary PCI is >250 s.

### 2.13 Following randomization

#### 2.13.1 Blinding

Blinding measures will be implemented. The participant will not be advised of treatment assignment. Conscious sedation will be maintained using midazolam and morphine to ensure patient comfort, as in standard care. The participant will be invited to listen to music of their choice using headphones. The effectiveness of blinding will be assessed by prospectively recording the participants' response to treatment group assignment.

### 2.14 Study assessments (Fig. 1)

Participants in both groups will be assessed with screening assessments of inclusion and exclusion criteria, ECG confirmation of anterior STEMI and measurement of left ventricular end-diastolic pressure (LVEDP).

**Fig. 1:**
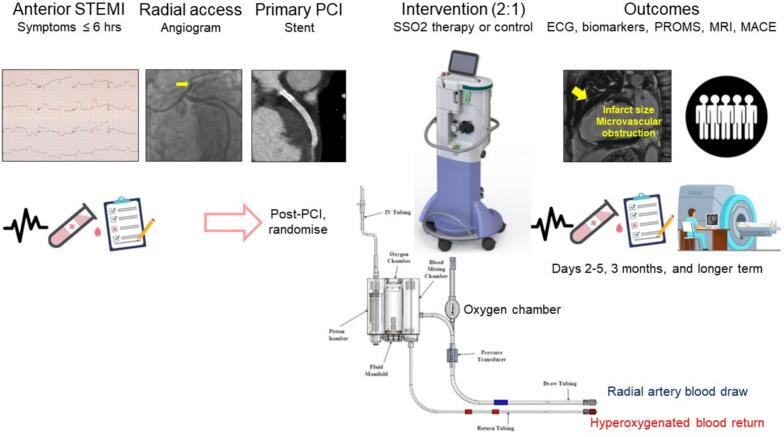
Flow diagram of the protocol.

Vital signs will be continuously monitored in the catheter laboratory as per standard care: blood pressure, heart rate, rhythm, blood pressure, %O_2_ saturation and ECG. ABGs and Killip Class will be checked at half hour intervals. Arterial blood gas samples provide PaO_2_ (inclusion criterion) and will be sampled before starting SSO_2_ therapy, and again at 30 and 60 min.

At the end of primary PCI, and again after SSO_2_ therapy or control, a limited (2 projection) coronary angiogram will be undertaken and hemodynamics (heart rate, invasive blood pressure and LVEDP) measured. Coronary microvascular function (index of myocardial resistance, coronary flow reserve, resistive reserve ratio and microvascular resistance reserve) will be assessed using a Pressure Wire X (Abbott Vascular, Santa Clara, California) placed in the left anterior descending coronary artery.

### 2.15 Intervention group

The intervention includes SSO2 therapy given for 1 h via a biradial extracorporeal circuit in association with intravenous glycoprotein IIbIIIa inhibitor therapy with tirofiban for up to 12 h.

Contralateral radial access will be established and a 6 or 7F sheath placed for blood draw at a rate of 100 mL/min. Radial artery access will involve passing an 0.035″ (135 cm) to provide support for positioning a longer guiding sheath with a hydrophilic distal coating e.g. Destination, Terumo (6–7 French, 65 cm length), with the distal end positioned in the axillary artery, distal to the origin of the vertebral and mammary arteries.

Blood will pass through the Downstream® System Console (ZOLL Circulation Inc); hyperbaric blood will be returned via the contralateral radial artery into the left main coronary artery using a 5 French diagnostic catheter (Impulse, Boston Scientific). SSO_2_ therapy will continue for 60 min. Therapeutic anticoagulation will be maintained with a target ACT >250 s, measured every 15–20 min. Intravenous glycoprotein IIb/IIIa inhibitor therapy will be given during SSO2 therapy and continued for up to 12 h afterwards.

### 2.16 Control group

Participants assigned to the control group will receive a mock (sham) experience. Additional radial artery access will not be obtained. Auto-perfusion is not proposed. The contralateral wrist will receive local anesthetic and manual pressure will be applied to mimic the experience of sheath insertion. A demonstrator function will be enabled on the console. Normal saline, rather than glycoprotein IIb/IIIa inhibitor, will be given intravenously for all controls and continued for up to 12 h.

### 2.17 Research blood sampling

These will be taken at baseline (before coronary angiogram, pre-PCI, pre-randomization, pre-SSO_2_), 75 min after initiation of SSO_2_ or control therapy, 24 h ± 4 h, 2–5 days (at the time of CMR imaging) and at 3 months (on attending for CMR scanning). Blood samples will be saved for future research analyses. High sensitivity troponin I (hsTnI) and NT-proBNP will represent surrogate outcomes for prognosis. Blood samples will be saved for future research analyses including measures of inflammation (hs-CRP, IL-6, ST2), vascular injury (ICAM, VCAM), myocardial infarction (hsTnI), coagulation (fibrinogen, vWF, tPA). A PAXGene tube will be collected for gene pathway analysis.

### 2.18 Coronary angiography and microcirculatory function tests

Analysis of angiographic images will proceed in a blinded manner using standardized core laboratory methods. Assessment of the following will be made at the end of primary PCI and following completion of treatment assignment: TIMI flow and thrombus grades in the culprit artery, as well as intraprocedural thrombotic events (IPTE), TIMI frame count and blush grade. Coronary microvascular function will be assessed, as above, in all patients at the end of standard care primary PCI and again following completion of treatment assignment using a Pressure Wire X (Abbott Vascular, Santa Clara, CA) placed in the left anterior descending coronary artery.

### 2.19 Cardiovascular magnetic resonance imaging

This will be conducted in line with standard clinical practice within 2–5 days of randomization and again at 3 months using a 1.5 Tesla scanner (Siemens MAGNETOM Avanto, Erlangen, Germany) and a standardized CMR protocol. The imaging protocol will include localizers, cine imaging for cardiovascular dimensions and function including long axis left ventricular imaging e.g., 4 and 3 chamber acquisitions, aortic cine and flow sequences, short-axis planes through the left ventricle (basal, mid-ventricular and apical) for T1-mapping, T2 and T2* mapping (mid-LV only) before administration of gadolinium contrast media, rest- perfusion imaging, short axis cine for LV function, then late gadolinium enhancement imaging and finally postcontrast myocardial T1 mapping. Infarct characteristics, including assessment for microvascular obstruction (MVO) and intramyocardial hemorrhage (T2* <20 ms) (binary yes/no and % LV mass) will be quantified.

Rest perfusion imaging of the 4-chamber and three short axis slices (base, mid and apex), will be acquired following intravenous injection of half-dose gadolinium-based contrast (Gadovist, Bayer) 0.05 mmol/kg 4 mL/s with 30 mL saline flush. Full-dose gadolinium contrast media will then be administered (Gadovist 0.15 mmol/kg at 4 mL/s with 30 mL saline flush) 10–15 min before late gadolinium enhancement imaging (3 long axis views, and short axis stack) acquired using phase-sensitive inversion recovery pulse sequence and free-breathing motion corrected technique.

#### 2.19.1 Stress perfusion CMR

This will be performed at 3 months follow-up. Patients will be asked to abstain from caffeine-containing beverages or foodstuffs for 24 h and vasoactive medications for 48 h prior to the CMR examination. Vasodilator stress will be achieved by intravenous infusion of adenosine at a dose of 140 μg/kg/min for 4 min (increased to 210 μg/kg/min for a further 2 min if no symptoms or <10 % heart rate increase). Splenic switch-off will retrospectively be confirmed during image analysis to assess for adequate stress. At peak stress, a gadolinium-based contrast agent (Gadovist, Bayer Healthcare) will be injected at 4 mL/s at a dose of 0.05 mmol/kg. Resting first-pass myocardial perfusion will be performed at least 10 min later.

### 2.20 CMR imaging analysis

Scans will be reviewed and reported by an imaging cardiologist blind to treatment group assignment using commercially available software (CVI42, Circle Cardiovascular Imaging, Calgary, Canada). Left ventricular (LV) volumes and function will be analyzed using manual planimetry. LV volumes at end-systole and end-diastole indexed to body surface area will be assessed at 2–5 days and again at 3 months. LV end-systolic volume index (LVESVI) is prespecified as a measure of LV remodeling and an increase in LVESVI at 3 months from baseline will be taken as a measure of adverse LV remodeling. Late gadolinium enhancement will be reported (17 segment model) with scores of 0 (no hyperenhancement), 1 (1 %–25 % extent), 2 (26 %–50 %), 3 (51 %–75 %), or 4 (>75 %). Size of myocardial infarction (% of LV mass) will be reported as will % myocardial salvage and the presence of myocardial hemorrhage. Incidental findings identified will be referred for interpretation by a radiologist and managed according to standard care. Automated quantitative pixel-mapping of myocardial blood flow will be performed using the Kellman Gadgetron framework supported by Siemens Healthcare.

### 2.21 Health status

These will be assessed using patient reported outcome measures (PROMS) during follow-up, including the EQ5D-5 L, Duke Activity Status Index (DASI) and Kansas City Cardiomyopathy (KCCQ) questionnaires.

### 2.22 Adjudicated adverse events

Follow-up assessments for adverse events will be performed by research staff blinded to baseline data and randomization. Contacts will involve in-person visits, telephone follow-up, or review of electronic health records. Clinical events identified as potentially relevant are to be assessed by a blinded Clinical Event Committee blinded to both baseline data and randomization, according to prespecified charter. The committee will be independent of the investigators, funder, and sponsor.

During the initial hospital stay, adverse events of interest will include peripheral and cerebrovascular ischemic events and bleeding events, defined by Bleeding Academic Research Consortium (BARC) [18].

Standardized cardiovascular and stroke endpoint definitions will be used [19]. Health outcomes will include death, and cardiovascular events including MI, heart failure, stroke/TIA, and bleeding. Data on health outcomes will be collected during index admission and after hospital discharge by clinical review for all patients at 3 months and by electronic health record checks at 12 months and at the close of the study (or by letter or patient review as appropriate to ensure complete data, including for quality of life). Electronic case record linkage using the Community Health Index (CHI) number in Scotland will allow tracking of all deaths and hospitalizations (and their causes). Functional status will be assessed using validated measures: EQ-5D-5L, DASI and KCCQ.

### 2.23 Assessment of health economics

An economic evaluation will compare usual care (as defined by the control group arm of the trial) with usual care-plus-SSO_2_ therapy. Resource use data will be collected for: days in hospital (including in each of the following ward types: intensive care, coronary care, and general cardiology ward), SSO_2_ therapy, medications given to the patient (e.g. glycoprotein IIb/IIIa), costs of procedures (including the duration of PCI, need for repeat procedures, use of implantable defibrillators and repeat hospital admissions related to the original STEMI event). Complications will be captured through added length of stay or through subsequent procedures carried out.

### 2.24 Follow-up

Patient follow-up will begin from the date the first patient is recruited until 12 months after the final patient is recruited.

### 2.25 Adverse events

All serious adverse events (SAE) and adverse events of interest will be prospectively assessed and documented for relatedness and severity. In addition, electronic case record linkage will be used to obtain information about health outcomes. These are quality assured systems made possible by electronic registration of all deaths and hospitalizations (and their causes).

#### 2.25.1 Periprocedural adverse events

Intra-procedural thrombotic events (IPTEs) are of interest and include new or worsened coronary thrombus, vessel closure, no reflow, slow flow and/or distal embolization. Peripheral ischemic events (cerebrovascular, peripheral) will also be documented.

#### 2.25.2 During initial hospitalization

Patients will be assessed as per study schedule by the research team for serious adverse events (SAEs), adverse events of interest (i.e. peripheral and cerebrovascular ischemic events) and bleeding events, defined by Bleeding Academic Research Consortium (BARC).

#### 2.25.3 During follow-up

Health outcomes will include death, and cardiovascular events including MI heart failure, stroke/TIA and bleeding. Data on health outcomes will be collected during the index admission and after discharge from each hospital by a clinic review for all patients at 3 months (including for the follow-up MRI scan) and by electronic health record checks at 12 months and at the close of the study (or by letter or patient review as appropriate to ensure complete data, including for quality of life).

### 2.26 Statistical considerations

Analyses will be descriptive, aiming to estimate between-group differences with 90 % confidence intervals. *P*-values will be reported, but the results will be interpreted in terms of the magnitude of estimated between-group differences, over time where applicable. Analyses will be according to intention-to-treat principles, supported by as-treated analyses. Missing data will not be imputed initially, but multiple imputation may be applied as sensitivity analyses.

### 2.27 Primary analysis and sample size calculation

The primary outcome is the within-participant change in the plasma concentration of NTproBNP measured at baseline, 24 h, days 2–5, and 3 months. The primary analysis will involve a between-group comparison of the log-transformed NTproBNP. In the MR-MI cohort study of 343 patients presenting with acute STEMI in the NHS Golden Jubilee hospital with follow-up assessments (cardiac MRI, NTproBNP) at 5 days and 6 months post-STEMI [4–6], a linear regression model of follow-up log NT-proBNP, adjusted for baseline, had a residual SD of 0.87. If a minimum of 51 randomized (2:1) participants have complete data, then with 80 % power and a 2-sided alpha of 0.05 the minimum detectable difference in SD units for the primary outcome is 0.85. To take account of incomplete SSO2 therapy and/or missing data for the primary outcome at baseline, then the minimum sample size is 56 patients, randomized 2:1 to the intervention versus control groups.

### 2.28 Secondary analyses

Between group differences in infarct size and myocardial salvage as quantitative traits (% of LV) will be assessed using appropriate general linear models adjusting for initial area-at-risk (% of LV). Other continuous outcomes, including biomarkers and coronary physiology parameters, will be analyzed in a similar fashion where the assumptions of linear modelling are sufficiently met, and adjusted for baseline data. Where data are clearly not normally distributed (e.g. laboratory variables) or modelling assumptions are not met, standard transformations will be applied to address these issues. The study is designed but not powered to assess secondary and clinical outcomes. In future analyses of clinical outcomes in the longer term, they will be presented with Kaplan-Meier time-to-event curves and compared where appropriate using hazard ratios and confidence intervals from Cox proportional hazards regression.

### 2.29 Trial management and governance

The study will be conducted in compliance with the Declaration of Helsinki, Good Clinical Practice and CONSORT guidelines [20].The study will be coordinated by the Study Management Group which will include those individuals responsible for the day-to-day management of the study including the Chief Investigator, Co-Investigators, and Research Nurse. Study monitoring will be conducted by monitors on behalf of NHS Golden Jubilee National Hospital Research Management Office. During monitoring assessments, Informed Consent Forms will be reviewed and source clinical data as appropriate.

### 2.30 Timeline

A 3-year timeline is initially proposed for enrolling the study population. Enrolment is anticipated to progress in phases from enrolment during office hours and then to extend towards enrolment out with office-hours, 24/7, as clinically appropriate.

## 3 Discussion

This prospective, randomized, sham-controlled and blinded trial will, first, test the feasibility of an exclusively radial approach to both the delivery of primary PCI, in line with clinical guidelines, and thereafter SSO_2_ therapy. In other regards, combination therapy with SSO_2_ and an intravenous glycoprotein IIbIIIa inhibitor will be delivered according to existing regulatory approvals.

The study design includes several novel features. First, this study will provide novel information on the effects of SSO2 therapy in patients with anterior STEMI using radial artery access. This novel approach for SSO2 therapy has not been previously described. The design involves a 2:1 randomization with the aim of maximizing the information on feasibility, safety and efficacy of SSO2 biradial therapy coupled with intravenous glycoprotein IIbIIIa inhibitor therapy to mitigate thrombus formation, as compared to a sham control group. The study will primarily provide information on feasibility and safety using radial artery access. This is important because femoral artery access was used in prior studies [4,5] whereas radial artery access is now recommended in clinical practice guidelines [8].

Second, by targeting a high-risk subset, patients with anterior STEMI and ischemic times of <6 h, the study will also provide novel data on the effect of SSO_2_ therapy on multisystem biomarkers of cardiovascular function. Specifically, detailed insights into treatment effect will further be provided by intracoronary physiology, CMR imaging (including estimation of absolute myocardial blood flow (mL/min/g) at 3-months using stress/rest cardiovascular MRI) and blood biomarkers (to provide multisystem insights into the potential mechanisms of any differences in infarct characteristics and LV remodeling).

Third, NTproBNP is selected as the primary outcome measure of efficacy since NTproBNP is validated for cardiac prognosis and the blood sampling approach permits serial assessments during a 3-month follow-up period, providing data on the within-subject change over time and to mitigate for the possibility of missing data on the primary outcome, should the participant not attend for a follow-up visit.

Increasing the number of sampling time points for the primary outcome mitigates the negative effects of missing data. For example, missing data are particularly problematic for paired evaluations (i.e. ×2, baseline and follow-up), which is the usual approach adopted for cardiac MRI, since missing data for just one sample rules-out paired comparisons, or any assessment of efficacy if no follow-up data are collected. Since the sample size of our trial is modest (*n* = 56), missing data would have a disproportionate effect, hence, this is mitigated by adoption of multiple sampling time points for the primary outcome.

Fourth, the final follow-up assessment is scheduled for 3-months. This time point takes account of the natural history of left ventricular remodeling post-MI (mostly established by 3-momths) balanced against the reducing likelihood of participant retention as follow-up is extended and increased costs of coordinating a clinical trial as the study duration is prolonged.

Finally, the fifth novel study design feature is participant blinding. This is achieved by implementation of a bespoke sham experience in the catheterization laboratory that is intended to mitigate the influence of bias on patient reported outcomes measures of health-related quality of life after treatment assignment. Data from this study are intended to inform a larger randomized study with clinical endpoints.

## CRediT authorship contribution statement

**Francis R. Joshi:** Writing – review & editing, Writing – original draft, Supervision, Project administration, Methodology, Investigation. **Maria Petty:** Project administration, Investigation. **Yen Wing Ng:** Investigation, Data curation. **Hamish Elliott:** Investigation, Data curation. **Ross Anderson:** Project administration, Methodology, Investigation. **Douglas Gordon:** Methodology, Investigation. **Rebecca Hanna:** Project administration, Data curation. **Robert Sykes:** Investigation, Data curation. **Shaun Leonard:** Project administration, Data curation. **Andrew Morrow:** Project administration, Investigation. **Dylan Tan:** Investigation, Project administration. **Anna Kamdar:** Data curation, Formal analysis, Investigation, Methodology. **Ramu Perumal:** Project administration, Methodology, Investigation, Funding acquisition. **Jeffrey L. Creech:** Resources, Project administration, Methodology, Investigation. **Peter Kellman:** Methodology, Investigation. **Paul Welsh:** Project administration, Methodology, Investigation, Formal analysis. **Alex McConnachie:** Formal analysis. **Colin Berry:** Writing – review & editing, Writing – original draft, Supervision, Software, Resources, Project administration, Methodology, Investigation, Funding acquisition, Data curation, Conceptualization.

## Funding

This study has been supported by 10.13039/100015345ZOLL Medical Corporation and the 10.13039/501100000274British Heart Foundation (RE/18/6134217).

## Declaration of competing interest

The author is an Editorial Board Member/Editor-in-Chief/Associate Editor/Guest Editor for this journal and was not involved in the editorial review or the decision to publish this article.

The authors declare the following financial interests/personal relationships which may be considered as potential competing interests: Francis Joshi has been paid for advisory work by Abbott Vascular, Shockwave Medical and Boston Scientific. He has also received research funding from Boston Scientific. CB is employed by the University of Glasgow, which holds consultancy and research agreements with companies that have commercial interests in the diagnosis and management of ischemic heart disease including Abbott Vascular, AskBio, AstraZeneca, Boehringer Ingelheim, CorFlow, Merck, Servier, Novartis, Roche, Siemens Healthcare, and Zoll Medical. Paul Welsh reports grant income from Roche Diagnostics, AstraZeneca, Boehringer Ingelheim, and Novartis, outside the submitted work, and speaker fees from Novo Nordisk and Raisio, outside the submitted work.

Ramu Perumal and Jeffrey L. Creech are employees of Zoll Medical.
